# Measurement of Ammonia and Hydrogen Sulfide Emission from Three Typical Dairy Barns and Estimation of Total Ammonia Emission for the Chinese Dairy Industry

**DOI:** 10.3390/ani13142301

**Published:** 2023-07-13

**Authors:** Zhifang Shi, Lei Xi, Xin Zhao

**Affiliations:** 1 College of Animal Science and Technology, Henan University of Animal Husbandry and Economy, Zhengzhou 450046, China; shizhifang83158@163.com (Z.S.); xilei@hnuahe.edu.cn (L.X.); 2 School of Ecology and Environment, Zhengzhou University, Zhengzhou 450001, China; 3 College of Animal Science and Technology, Northwest A&F University, Yangling 712100, China; 4 Department of Animal Science, McGill University, 21111 Lakeshore Road, Sainte-Anne-de-Bellevue, QC H9X 3V9, Canada

**Keywords:** ammonia emission, hydrogen sulfide emission, environmental contamination, dairy farms

## Abstract

**Simple Summary:**

Ammonia (NH_3_) and hydrogen sulfide (H_2_S) are obnoxious gases with characteristic odors. They are environmental contaminants and could be harmful to animals and humans. Measurement of NH_3_ and H_2_S from dairy farms is still limited, especially for Chinese dairy farms. In this study, we measured ammonia and hydrogen sulfide from 11 barns from 3 dairy farms in central China during winter. Next, we calculated the average daily emission rates for these two gases. Finally, we extrapolated our results to estimate the NH_3_ emission from the Chinese dairy production.

**Abstract:**

There is an urgent need for accurate measurement for emissions of ammonia (NH_3_) and hydrogen sulfide (H_2_S) in dairy barns in order to obtain reliable emission inventories and to develop and evaluate abatement strategies. This experiment was performed on three dairy farms in central China during 14 consecutive days in the winter 2020. Concentrations of NH_3_ and H_2_S were measured every two hours. The samples were taken inside and outside of barns from 7 sites at two heights (at floor and 1.5 over the floor). The results show that the average NH_3_ concentration was 2.47 mg/m^3^ with a maximum of 4.62 mg/m^3^, while the average H_2_S concentration was 0.179 mg/m^3^ with a maximum of 0.246 mg/m^3^. Lactating cows produced significantly more NH_3_ (3.73 mg/m^3^ versus 2.34 mg/m^3^) and H_2_S (0.24 mg/m^3^ versus 0.14 mg/m^3^) than non-lactating cows. NH_3_ and H_2_S concentrations were higher at 0 m than at 1.5 m, especially during the day. In addition, the average daily emission rates per animal unit (AU = 500 kg weight) were 23.5 g and 0.21 g for NH_3_ and H_2_S, respectively. The emission rate for NH_3_ was then used to extrapolate the NH_3_ emission from the Chinese dairy production. Our estimation for 2016 was 0.45 Tg, and it could reach 1.35 Tg by 2050. These numbers reflected our first attempt to calculate emission inventories for the Chinese dairy industry. Our results also suggest that more concrete measures must be taken to reduce the uncertainties of NH_3_ emissions from dairy cow production in China.

## 1. Introduction

Environmental issues are one of the major factors affecting the sustainability of the dairy industry, in addition to social and economic issues. Dairy cows and their manure emit greenhouse gas and other air pollutants which contribute to climate change [1]. While much research has been focused on greenhouse gases in recent years, much less has been focused on other air pollutants. Ammonia (NH_3_) is quantitatively the largest emitted gas from dairy production, while other air pollutants include reactive nitrogen species (e.g., nitrogen oxides and nitrous oxide), odor emissions (e.g., organic acids), and gaseous sulfur compounds (e.g., hydrogen sulfide (H_2_S)). NH_3_ can have significant effects on the natural environment, mainly through the acidification and eutrophication of ecosystems. Recently, the potential effect of NH_3_ on human health has been raised as a concern. Atmospheric NH_3_ is a key precursor to neutralize H_2_SO_4_ and HNO_3_ in the air and form (NH_4_)_2_SO_4_, NH_4_HSO_4_, and NH_4_NO_3_ [2,3], which contribute to reduced visibility, regional haze and health impacts associated with fine particular matters such as PM_2.5_. In China, the most concerned adverse impact of NH_3_ is that it can facilitate the formation of PM_2.5_ [4]. It has been suggested that abating NH_3_ was more cost-effective than nitrogen oxides for mitigating PM_2.5_ air pollution [5].

NH_3_ and H_2_S are released from dairy manure due to the inefficient conversion of dietary nitrogen to animal products and decomposition of sulfur-containing organic compounds in the manure under anaerobic conditions, respectively. Emission of NH_3_ also represents a significant loss of dietary crude proteins. It has been estimated that total N output via milk and meat was only 11% of total N input for dairy cows [6]. The excreted N is lost at each stage of the manure management chain (e.g., during collection, storage, and after land application of manure) in several forms: NH_3_, nitrous oxide, and nitrate [7]. NH_3_ volatilization is the major form of N losses to the environment and accounts for 15 to 50% of the excreted manure N [8]. In a case study specific to the northeast dairy region of the United States, Rotz et al. [9] estimated that NH_3_ emission was the greatest concerns for the Pennsylvania dairy industry because it was responsible for more than half of the state’s emissions. The same study also suggested focusing on NH_3_ abatement to reduce overall reactive N (e.g., NH_3_, nitrous oxide, nitrate, and other forms of gaseous nitrogen oxides) losses from the dairy production system. In addition, NH_3_ is an important biotoxic and neurotoxic substance [10]. Economically, NH_3_ emission from ruminant production in China was estimated to cost $21.7 billion to human health and $0.34 billion to ecosystems [11]. Finally, globe trading of milk products can transfer significant amounts of environmental impacts from importing countries to exporting countries. For example, China’s import of ruminant products and livestock feed in 2012 transferred 42.8 Gg NH_3_ emissions to exporting nations [11].

H_2_S is produced as manure decomposes anaerobically, resulting from the mineralization of organic sulfur compounds, as well as the reduction in oxidized inorganic sulfur compounds such as sulphate by sulfur-reducing bacteria [12]. H_2_S produced can be both an odor nuisance and a health hazard, even at low concentrations. The amount of H_2_S co-emitted with NH_3_ from livestock production is much less than that of NH_3_, even though H_2_S is the major sulfur compound emitted from livestock production [13,14]. Data on emissions of H_2_S from dairy production are still relatively scarce. Our previous study [15] was among the few that measured H_2_S concentrations in dairy barns. However, estimation of the total H_2_S emission from the dairy production is still difficult, since many basic data required for estimation are still missing.

To control and reduce emission of NH_3_ and H_2_S from dairy operations, there will be a continued need for emission inventories based on reliable and representative emission rates, as well as an increasing demand for mitigation strategies. In general, there is a lack of information on NH_3_ and H_2_S emissions from dairy barns in Asian countries, with most published data deriving from Europe and North America. In a previous study, we have reported that NH_3_ and H_2_S concentrations in dairy barns were significantly correlated with nitrogen and sulfur contents in feed and manure as well as with temperature inside the barns [15]. In addition, we calculated and compared the emission rates for NH_3_ and H_2_S with published ones from other countries. However, the previous study was only carried out in summer. It is well known that seasons affect NH_3_ and H_2_S emissions from cattle operations [16], in addition to other factors such as manure handling practices (scraping or flushing), manure removal frequency, floor types (solid or slatted), barn types (free-stall or tie-stall), ventilation of barns, and wind speed [17]. Except those in the Northeastern region, dairy barns in most parts of China have walls which can be open or closed. The wall is open during summer and is covered by curtains during winter. Therefore, this study was undertaken to quantitatively measure NH_3_ and H_2_S emission from the dairy barns during winter and calculate the emission rates for NH_3_ and H_2_S. Henan Province is one of the major dairy production areas in China, with typical temperate climate characteristics. The dairy barn structure used in our study is typical for most Chinese dairy regions. Another aim of this study was to use the emission rates for NH_3_ generated in this study and our previous study [15] for estimating NH_3_ emission for the recent (2016) and future (2050) Chinese dairy industry.

## 2. Materials and Methods

### 2.1. Site and Building Description

**Figure 1:**
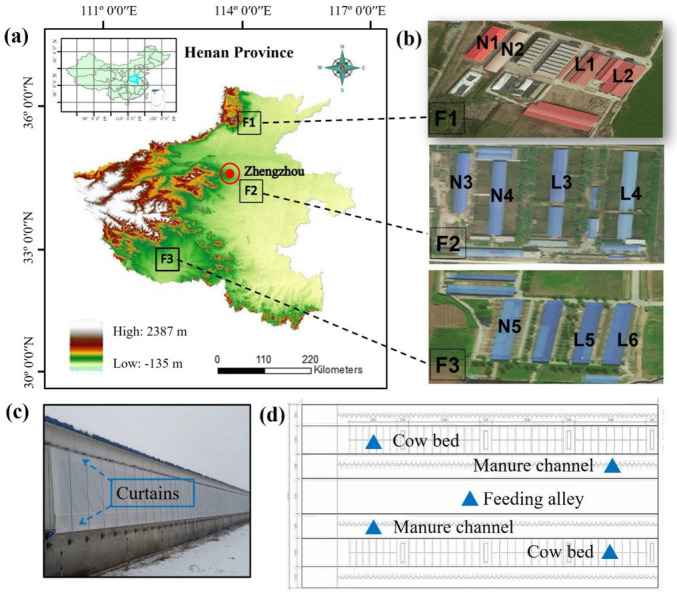
Henan Province is one of the major dairy production areas in China, with Zhengzhou as its capital (**a**). Eleven barns on the three farms were used for this experiment. F1, F2, and F3 farms are in Hebi city, in Zhengzhou city, and in Sanmenxia city, respectively (**b**). Two barns for lactating cows (L1, L2) and two barns for non-lactating cows (N1, N2) on F1, two barns for lactating cows (L3, L4) and two barns for non-lactating cows (N3, N4) on F2, and two barns for lactating cows (L5, L6) and one barn for non-lactating cows (N5) on F3. In winter, each barn is covered with curtains (**c**). There were five monitoring points in each dairy barn, two manure channel sites, one feeding alley site, and two cow bedding sites (**d**).

The three commercial dairy farms used in this study (F1, F2, and F3 farms) are in the Henan province in central China (Figure 1) and they were also used in our previous study [15]. The prevailing winter wind for the regions was northwest wind during winter. Eleven barns on the three farms were used for this experiment: two barns for lactating cows (L1, L2) and two barns for non-lactating cows (N1, N2) on F1, two barns for lactating cows (L3, L4) and two barns for non-lactating cows (N3, N4) on F2, and two barns for lactating cows (L5, L6) and one barn for non-lactating cows (N5) on F3. Average body weights of lactating dairy cows were 670 kg, 630 kg, and 650 kg, respectively, and average body weights of non-lactating dairy cows were 652 kg, 639 kg, and 647 kg, respectively, for F1, F2, and F3 farms. There were 391, 342, and 288 lactating cows, and 216, 248, and 216 dry cows, on F1, F2, and F3 farms, respectively. The average milk yields of dairy cows in F1, F2, and F3 farms were 31 kg, 26 kg, and 26 kg, respectively. The open walls for all 11 barns were covered by insulation curtains in winter (Figure 1). Thus, these barns could be treated as naturally ventilated closed barns. There were outdoor exercise areas for all three farms.

### 2.2. Experimental Periods, Production and Feed

All experimental protocols used in this experiment were approved by the Henan University of Animal Husbandry and Economy Institutional Animal Care and Use Committee (protocol number HNUAHE 466) and all the institutional safety procedures were followed during the experimentation. Measurements were conducted over a 2-week period in the winter of 2020. The total sampling period for each barn was 48 h. All cows were machine-milked three times daily at 4:00, 14:00, and 21:00. All cows were fed three times daily at 8:30, 12:00, and 18:00 with total mixed ration (TMR). Manure was removed twice daily at 8:00 and 17:00 with a bulldozer. TMR was used for both non-lactating dairy cows and lactating cows. Feed nitrogen content and feed sulfur content of the TMR were 3.04% and 0.26% for lactating dairy cows, and 2.76% and 0.23% for non-lactating cows. The average feed consumption was 45.5 kg/cow per day for lactating cows, and 40.4 kg/cow per day for non-lactating cows, respectively.

### 2.3. Measurement of Gas Concentrations and Environmental Parameters

Gas concentrations and environmental parameters were measured both inside (two manure channel sites, one feeding alley site, and two cow bedding sites) and outside (two locations 20 m away from the barn as a blank) the barns (Figure 1). Samples were taken from these seven sites both at the height of either near the floor (0 m) or 1.5 m above the floor. Wind speed, atmospheric pressure, CO_2_, and total suspended particles (TSP) were measured every two hours, while the temperature and humidity were recorded every 5 min, using an automatic temperature and humidity recorder (LGR-WSD20, Rogue Instrument Ltd., Hangzhou, China). An anemometer (405-V1, Testo, Lenzkirch, Germany), a barometer (DYM3 Yipin Instrument Ltd., Shanghai, China), a portable CO_2_ detector (JSA8, Jiada Instrument Ltd., Shenzhen, China), and a dust detector (JC-1000, Jingcheng Instrument Ltd., Qingdao, China) were adopted to measure wind speed, atmospheric pressure, CO_2_, and TSP, respectively.

NH_3_ and H_2_S were collected and measured every two hours. These two gases were collected using an integrated air sampler (2000C, Tuowei Instrument Ltd., Qingdao, China, flow range 0.1 L/min–1.0 L/min). NH_3_ was measured by the Nessler′s reagent spectrophotometry method [18], with the detection limit of NH_3_ of 0.01 mg/m^3^. In addition, the H_2_S content was measured using the methylene blue spectrophotometric method [18] with minor modifications [15], with the minimum detectable concentration of 0.001 mg/m^3^. A spectrophotometer (C752N754PC, Jinghua Instrument Ltd., Shanghai, China) was used for colorimetric analyses of both NH_3_ and H_2_S concentrations.

### 2.4. Calculation of Ventilation Rate and Emission Rate

The CO_2_ balance method [19,20] was used to calculate the ventilation rate. Assuming ideal mixing with the air inside the building, the relationship between the ventilation rate and the gas production rate can be estimated using Equation (1).

(1) $$Q=\frac{N\cdot P_{{CO}_{2}}}{C_{i}-C_{o}}$$

where Q is the ventilation rate (m^3^/h); N is the number of cows housed inside the building; *P*_CO_2__ represents the excretion rate of CO_2_ from one cow (g/cow/h); C_i_ and C_o_ are the average concentrations of the gas inside and outside the building, respectively (g/m^3^);

The emission rate of a gas was calculated using the following Equation (2).

(2) $$E_{t}=Q(C_{i}-C_{o})$$

E_t_ is the mission rate of a gas (g/h), while Q is the ventilation rate (m^3^/h) from the previous calculation. C_i_ and C_o_ have been defined as before for Equation (1).

The emission was also calculated per animal unit (AU) instead of emission per cow. The AU is equivalent to 500 kg animal mass [21]. The emission rate per AU can thus be stated as Equation (3).

(3) $$E=\frac{E_{t}\times 500}{N\times m}$$

E is the gas emission rate per animal unit (g/AU/h), while E_t_ is the emission rate of a gas (g/h). N represents the total number of cows housed inside the building, while m is the average mass of a cow accommodated in the building (kg/cow).

### 2.5. Data Analyses

Data analysis was performed using SPSS statistical software (version 23; SPSS Inc., Chicago, IL, USA). Data were analyzed using one-way ANOVA with Least-Significant Difference (LSD) multiple comparisons. The significance was declared at *p* < 0.05. The graphs were created using Prism software (GraphPad, San Diego, CA, USA).

A mixed linear model was used to describe the effect factors on NH_3_ or H_2_S concentrations as equation Y*ijkl*= µ + S*i* + P*j* + H*k* + T*l* + S*i* × T*l* + *εijkl*, where Y*ijkl* = the dependent variable, µ= general mean, S*i* = the effect of site (*i* = 1, 2, 3), P*j* = the effect of stage of productivity (*j* = 1, 2), H*k* = the effect of measuring height (*k* = 1, 2), T*l* = the effect of time (*l* = 1, 2, 3), *S*i × T*l* = Effect of the interactions between site and time, and ε*ijkl* = the residual effect. All other effect factors and their interactions were also considered during the initial stage and were removed from the model due to insignificant effects.

## 3. Results and Discussion

### 3.1. Environmental Parameters

As shown in Table 1, the temperature, the relative humidity, and CO_2_ concentrations were significantly higher inside the barn than those outside the barn by 4.1 °C, 18.9%, and 120 mg/m^3^, respectively (*p* < 0.05). Indoor air speed was significantly lower than that of the outdoor by 1.41 m/s (*p* < 0.05). There was no significant difference in air pressure and TSP between inside and outside of the dairy barns.

### 3.2. Concentrations of NH_3_ and H_2_S in the Barns

**Figure 2:**
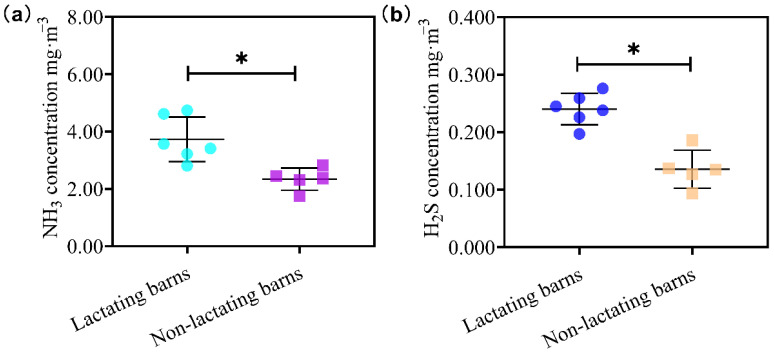
The comparison of NH_3_ (**a**) and H_2_S (**b**) concentrations between lactating dairy barns and non-lactating dairy barns. The solid circle (●) represents the average concentration for each lactating dairy barns (L1 to L6), and the square (■) represents the average concentration for each non-lactating dairy barns (N1 to N5). The data were measured every 2 h for 48 h. The average NH_3_ or H_2_S concentration in each dairy barn is the average of 120 monitoring values (*n* = 120). The long horizontal line is for the means of lactating or non-lactating bans, while the short horizontal lines are for the standard deviation (SD) of lactating or non-lactating bans. The star (*) indicates a significant difference between lactating and non-lactating barns (*p* < 0.05).

**Figure 3:**
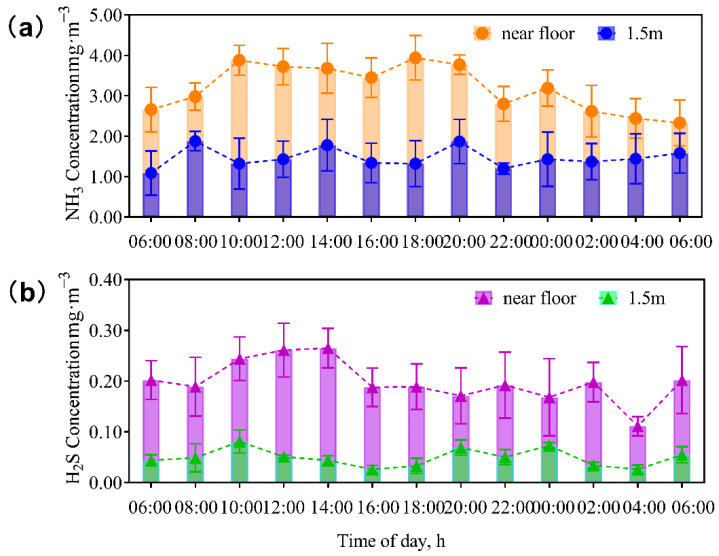
Diel changes in NH_3_ (**a**) and H_2_S (**b**) inside the dairy barns in the winter. The concentrations of NH_3_ and H_2_S were the mean ± standard deviation (SD) of 11 dairy barns (*n* = 110). For each barn, NH_3_ and H_2_S was measured at five sites (two manure channel sites, one feeding alley site, and two cow bedding sites) as indicated in Figure 1. Each location was sampled both near the floor and at 1.5 m above the floor. Measurements were made every two hours for 48 h. The values for NH_3_ and H_2_S represent the average of each sampling time point for each height.

The overall average (11 barns) concentrations of NH_3_ ranged from 1.69 to 4.62 mg/m^3^, while the average concentrations of H_2_S oscillated between 0.094 and 0.246 mg/m^3^. Lactating cows produced significantly more NH_3_ (3.73 mg/m^3^ vs. 2.34 mg/m^3^) and H_2_S (0.24 mg/m^3^ vs. 0.14 mg/m^3^) than non-lactating cows (Figure 2). As shown in Figure 3, NH_3_ and H_2_S concentrations were higher at 0 m than at 1.5 m, especially during the day. In addition, the diel pattern was obvious only for measurements near floor, not for those at the height of 1.5 m. At the floor level, the concentrations of NH_3_ were higher between 10 a.m. and 8 p.m. than those during the night, while the concentrations of H_2_S were higher between 10 a.m. and 2 p.m. than those during the night.

The current study measured the NH_3_ and H_2_S concentrations in typical dairy barns in central China. The average NH_3_ concentration was 2.47 mg/m^3^, while the average H_2_S concentration was 0.179 mg/m^3^. In comparison, the average NH_3_ concentration was 1.54 mg/m^3^, while the average H_2_S concentration was 0.092 mg/m^3^ on these same farms in the summer [15]. NH_3_ and H_2_S concentrations in winter were 60.4% and 94.6% higher than those in summer, respectively. The seasonal effects on NH_3_ and H_2_S production and emission depend on both temperature effects and the housing effects. The temperature effect can be explained by the activities of enzymes involved in the chemical reactions. The urease activity is optimum at 35 °C and declines with decreasing temperature, as shown by Hao [22]. Thus, low temperature during the winter would reduce NH_3_ production. Specific enzymes involved in H_2_S production by bacteria in manure are less clear, but it is expected that reduced barn temperature during the winter could also reduce the enzyme activities and produce less H_2_S. Our assumption was supported by Dai and Karring [23], who reported a significant relationship between temperature in the barn and NH_3_ production. There was a difference of 21 degrees in the average barn temperature between the winter in this study and the summer in our previous study [15]. Thus, the production and valorization of NH_3_ and H_2_S are expected to be decreased during the winter [24]. On the other hand, the walls of the same barns were open and the spray + fan cooling systems were functional during summer [15]. During winter, closed walls by curtains and closing of the spray + fan cooling system reduced the dissipation of NH_3_ and H_2_S out of the barns, which could be the main reason for the higher concentrations during winter. These changes were reflected in the wind speed in the barns. The wind speed in the barn was 1.81 m/s during summer and 1.36 m/s during the winter. Thus, the higher concentrations of NH_3_ and H_2_S during the winter reported in this study than those during the summer [15] was not due to higher production of these two gases, rather due to less dissipation of them from the barns. Our conclusion was supported by Saha et al. [16], who confirmed that the NH_3_ concentration and its discharge rate in naturally ventilated barns were affected by wind speed.

Concentrations of NH_3_ and H_2_S registered in this study did not exceed the current Chinese governmental regulations on these toxic gases, which are NH_3_ ≤ 20 mg/m^3^ and H_2_S ≤ 8 mg/m^3^, respectively, for the dairy barns [25]. In comparison, the EU stipulates 20 ppm (14 mg/m^3^) for occupational exposure to NH_3_, while France and Norway set the limit at 10 ppm (7 mg/m^3^) and 25 ppm (17.5 mg/m^3^), respectively [26]. The US national institute of occupational safety and health (NIOSH) specifies that the maximum human exposure concentration to NH_3_ is 25 ppm (17.5 mg/m^3^) during 8 h or 35 ppm (24.5 mg/m^3^) for 15 min. Long-term exposure to NH_3_ even at the level of 0.3 mg/L will endanger human and animal health [27]. Considering that the most concerned adverse impact of NH_3_ is on its facilitation in forming PM_2.5_ in China [4] and other countries such as USA [28], it is still prudent to monitor and reduce NH_3_ and H_2_S concentrations in the barns, since poor manure management, inadequate ventilation and high room temperature can lead to high gas concentrations [29], which is harmful to farm workers, dairy cows, and the environment.

### 3.3. Emission Rates of NH_3_ and H_2_S

**Figure 4:**
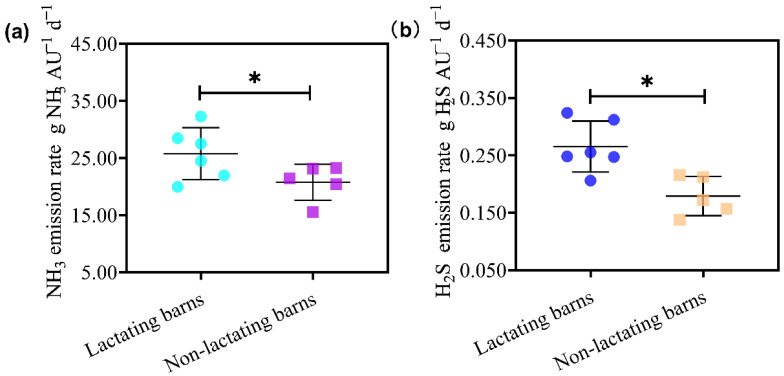
The comparison of emission rates for NH_3_ (**a**) and H_2_S (**b**) between lactating dairy barns and non-lactating dairy barns in the winter. The solid circle (●) represents the average for each lactating dairy barns (L1 to L6), and the square (■) represents the average for each non-lactating dairy barns (N1 to N5). There are five monitoring points in each dairy barn, and the data are measured every 2 h for 48 h of continuous monitoring. The average NH_3_ or H_2_S emission rates in each dairy barn is the average of 120 monitoring values (*n* = 120). The long horizontal line is for the means of lactating or non-lactating bans, while the short horizontal lines are for the standard deviation (SD) of lactating or non-lactating bans. The star (*) indicates a significant difference between lactating and non-lactating barns (*p* < 0.05).

The overall average (11 barns) emission rates of NH_3_ ranged from 15.52 to 34.75 g NH_3_/AU/d, while emission rates of H_2_S ranged from 0.137 to 0.312 g H_2_S/AU/d. The average NH_3_ emission rate was 23.5 g NH_3_/AU/d, while the average H_2_S emission rate was 0.21 g H_2_S/AU/d. The average of NH_3_ emission rate was 26.79 g NH_3_/AU/d, while the average emission rate of H_2_S was 0.27 g H_2_S/AU/d for lactating dairy cows. On the other hand, the average of NH_3_ emission rate was 18.45 g NH_3_/AU/d and the average emission rate of H_2_S was 0.18 g H_2_S/AU/d for non-lactating dairy cows (Figure 4). The higher emission rates of NH_3_ and H_2_S for lactating cow barns than those for the non-lactating cow barns may be related to the higher feed intake for lactating cows.

Emissions rates of NH_3_ and H_2_S for the Chinese dairy industry were lacking, even though China is the third largest country for dairy production [30]. The average emission rates of NH_3_ and H_2_S were 23.5 g/AU/d and 0.21 g/AU/d, respectively, in this study. As a comparison, the average emission rates of NH_3_ and H_2_S for the summer were 26.8 g/AU/d and 0.26 g/AU/d for the summer [15]. Lower emission rates for the winter versus summer reported in this study are in agreement with those in the literature. Bougouin et al. [31] carried a meta-analysis of NH_3_ emission and found that season significantly affected NH_3_ emission rates. The average NH_3_ emission rate was 59.7 g/cow/d for the winter (*n* = 26) and 91.7 g/cow/d for the summer (*n* = 29). Our NH_3_ emission rates were much lower than most reported emission rates, even after consideration of different units used in our study and the meta-analysis study by Bougouin et al. [31]. This discrepancy could be caused by relatively poor nutrition for our dairy cows, reflected by low milk production levels. Very limited reports are available for the seasonal effect on H_2_S emission rate. Maasikmets et al. [32] from Estonia reported 0.14 g/AU/d for the winter, much lower than our numbers. Mazur et al. [33] showed that temperature affected NH_3_ emissions. In this study, the emission rates of NH_3_ and H_2_S decreased, respectively, by 12.1% and 19.2% in the winter in comparison with the summer. The same diets and same manure management system were used on the 11 dairy farms all year around and they were not contributing factors for the seasonal effects on our farms. Therefore, the temperature in the barn might be responsible for our observed differences in the emission rates during two seasons.

### 3.4. Estimation of NH_3_ Emission from Chinese Dairy Industry

**Figure 5:**
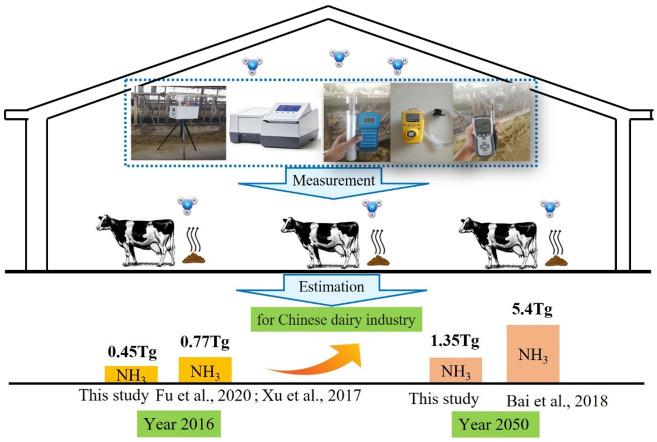
Prediction of ammonia emissions from dairy farming. The results of the study by Fu et al. [36], Xu et al. [37] and Bai et al. [6] were used for estimation and comparison.

The estimated emission rates from this study and our previous study [15] have allowed us to calculate the emission of NH_3_ from Chinese dairy barns today and in the future. In 2016, there were 12.72 million dairy cows in stock in China [30]. Our assessment was focused on NH_3_ emission from dairy barns. The major sources of NH_3_ emissions in a typical dairy operation are the barns, the manure storages (mainly anaerobic lagoons), and from field application of manure. We recognize that it is very difficult to extrapolate the emission data from dairy barns to the total NH_3_ emission from the dairy production. Through a comprehensive review of NH_3_ emissions from dairy housing facilities, Hristov et al. [34] reported an average daily emission of 59 g NH_3_/cow. On the other hand, Rotz et al. [35] estimated that NH_3_ emission from dairy cows were 227 g NH_3_/day per cow, including all farm sources of NH_3_ emission. Assuming that the ratio between the two numbers from the two previous references was correct and that all cows in China were housed, we estimated that the NH_3_ emission from Chinese dairy barns would be 0.45 Tg (0.45 Tg = 12.72 million cows × (23.5 g NH_3_/AU/d for winter + 26.8 g NH_3_/AU/d for summer)/2 × 227/59 × 365 days) in 2016. Fu et al. [36] estimated by a model that the total ammonia emissions in China was 11 Tg in 2016. Assuming that cow production emissions accounted for 7% of the total ammonia emissions [37], ammonia emissions from the Chinese dairy were estimated about 0.77 Tg in 2016. Similarly, it is projected that milk consumption in China will increase from current of 31 kg per capital to 82 kg per capital in 2050 [6]. Thus, the emission of both NH_3_ and H_2_S from dairy production is expected to increase if the production efficiencies are not significantly improved. According to the forecast of FAO, China’s population will reach 1.4 billion by 2050 [30]. Based on these predications, China’s milk demand will reach 114.8 million tons in 2050, which is three times as much as in 2016. Assuming no changes in productivity from the current level, NH_3_ emissions from dairy cow production could reach 1.35 Tg by 2050, according to our estimation (Figure 5). On the other hand, Bai et al. [6] estimated by using a model that total reactive nitrogen loss from dairy production in China could reach 5.4 Tg in 2050. This amount equals 3.46 Tg of ammonia, assuming that 64% of the emitted reactive N was in the form of NH_3_ [33]. While we recognize that our calculations were based on several assumptions which may not be totally true, it seems that Bai et al. [6] and Fu et al. [36] may have overestimated ammonia emissions for the Chinese dairy production. In addition, we recognize the limitation that our emission rates came from regional dairy farms, rather than dairy farms from all dairy regions in China. Thus, more direct measurement of NH_3_ emission is needed for improved accuracy. However, these estimations are important for creating baselines, establishing mitigation goals, and generating strategic regional and national policies for sustainable dairy development. In any case, the estimations by others and our data call for more effective mitigation measures to reduce the emission of NH_3_ from the dairy industry.

## 4. Conclusions

In summary, emission of NH_3_ and H_2_S was measured and estimated from 11 barns from 3 dairy farms in central China during winter. The average NH_3_ concentration was 2.47 mg/m^3^ with a maximum of 4.62 mg/m^3^, while the average H_2_S concentration was 0.179 mg/m^3^ with a maximum of 0.246 mg/m^3^. In addition, the average daily emission rate for animal units were 23.5 g and 0.21 g for NH_3_ and H_2_S, respectively. Lactating dairy cows had higher NH_3_ and H_2_S emission rates compared to non-lactating dairy cows due to higher feed intake and higher forage nitrogen content. According to our estimation, the NH_3_ emission from the Chinese dairy production was 0.45 Tg in 2016, and it could reach 1.35 Tg by 2050. These results call for more effective mitigation measures to reduce the emission of NH_3_ from the Chinese dairy industry. In addition, increasing dairy cow population is not the best strategy for the development of Chinese dairy industry. More efforts should be devoted to improving the production efficiency and adopting new measures for environmental control for the healthy development of the dairy industry.

## Figures and Tables

**Table 1:** The average values of environmental parameters in the winter.

Environment Parameters ^1^	Indoor					Outdoor				
	Mean ^2^	SD	CV/%	Min	Max	Mean ^2^	SD	CV/%	Min	Max
Temperature (°C)	6.2 ^a^	3.3	78.6	2.6.5	12.1	2.1 ^b^	0.7	33.3	−6.2	9.7
Relative humidity (%)	69.1 ^a^	6.9	14.4	58.0	79.5	50.2 ^b^	10.3	20.5	55.0	80.1
Wind speed (m/s)	1.36 ^b^	0.31	22.8	0.49	1.94	2.77 ^a^	1.01	36.5	1.25	3.90
CO_2_ (mg/m^3^)	509.0 ^a^	88.5	17.4	422.1	548.1	389.0 ^b^	44.5	11.4	350.7	431.1
Air pressure (kPa)	98.8	1.9	2.0	96.6	99.8	96.5	1.1	1.14	92.6	97.8
TSP ^3^ (mg/m^3^)	0.288	0.045	15.6	0.147	0.269	0.242	0.043	12.6	0.163	0.245

^a,b^ Means with different letters within the same row are significantly different (*p* < 0.05); ^1^ For each barn, environmental parameters were measured at two outside locations (two upwind blank areas 20 m away from the barn) and five inside locations. Each inside location was sampled both near the floor and at 1.5 m above the floor. Measurements were made every 2 h for 48 h; ^2^ Means in the table were the average of all values at two heights during the experiment; ^3^ TSP stands for total suspended particles.

## Data Availability

The data presented in this study are available upon request from the corresponding author.
